# Absolute Income, Income Inequality and the Subjective Well-Being of Migrant Workers in China: Toward an Understanding of the Relationship and Its Psychological Mechanisms

**DOI:** 10.3390/ijerph16142597

**Published:** 2019-07-21

**Authors:** Kaizhi Yu, Yun Zhang, Hong Zou, Chenchen Wang

**Affiliations:** 1 School of Statistics, Southwestern University of Finance and Economics, Chengdu 611130, China; 2 School of Economics, Southwestern University of Finance and Economics, Chengdu 611130, China; 3 School of Arts, English and Languages, Queen’s University Belfast, Belfast BT7 1FY, UK

**Keywords:** subjective well-being, migrants, absolute income, income inequality

## Abstract

No study has been conducted linking Chinese migrants’ subjective well-being (SWB) with urban inequality. This paper presents the effects of income and inequality on their SWB using a total of 128,000 answers to a survey question about “happiness”. We find evidence for a satiation point above which higher income is no longer associated with greater well-being. Income inequality is detrimental to well-being. Migrants report lower SWB levels where income inequality is higher, even after controlling for personal income, a large set of individual characteristics, and province dummies. We also find striking differences across socio-economic and geographic groups. The positive effect of income is more pronounced for rural and western migrants, and is shown to be significantly correlated with the poor’s SWB but not for the well-being of more affluent respondents. Interestingly, high-income earners are more hurt by income inequality than low-income respondents. Moreover, compared with migrants in other regions, those in less developed Western China are found to be more averse to income inequality. Our results are quite robust to different specifications. We provide novel explanations for these findings by delving into psychological channels, including egalitarian preferences, social comparison concerns, expectations, perceived fairness concerns and perceived social mobility.

## 1. Introduction

China has been witnessing a surge of internal labor migration since the late 1980s. The latest official figures estimate the total number of migrant workers in 2017 at 244 million [1]. Some are urban-urban migrants, but the vast majority is rural-to-urban migrant workers accounting for more than 70% of the total number of migrants [2]. With an increasing number of migrants temporarily or permanently settling in host cities, migrants will inevitably become a significant proportion of the urban population. As the case in Shenzhen, migrants have even exceeded urban local residents [3]. Related to this massive inflow, understanding how these huge migrants feel about their lives and what drives their subjective well-being (hereafter called SWB or happiness) in destination cities has attracted increasing attention from both scholars and policymakers.

Migration theory posits that migrant workers leave their hometown to pursue better jobs and higher income, which further provide a means to maximize their SWB [4,5]. The bulk of the research has suggested that migrants generally find adjusting to life in their host cities to be challenging [6,7]. Migrants tend to have a lower level of SWB than local residents, as they have to get accustomed to their new social circumstance and build up new social ties after their arrivals [8,9]. Migrants’ false aspirations also contribute to their unhappiness [10]. Migrants may not realize that their aspirations, which are influenced by reference groups from new surroundings, have risen along with income and have exceeded their actual achievements. Another stream of literature has argued that migrants who have achieved upward social mobility in the host city would experience an increase in SWB due to improvements in socio-economic status [11,12]. The phenomenon of rural-to-urban migration in China has been totally different from that in North America, Europe, and other developing countries because of its unique dualistic economic structure, household registration (*hukou*) system, and sharp urban-rural divide in income [13,14]. In recent years, the socio-economic status of Chinese migrants has distinctly improved, including income, employment, living conditions, and welfare [15,16]. Despite much attention paid to migrants’ observable socio-economic achievements [17,18,19], it is still unknown whether attaining higher socio-economic status, particularly income, actually leads to increased SWB.

During China’s rapid urbanization, income inequality has become increasingly serious [20,21]. At the early stage of economic reform in the 1980s, China’s Gini coefficient was roughly 0.3 [22]. It was estimated by the National Bureau of Statistics that the Gini coefficient reached a high level of 0.467 in 2017. Income inequality has become a severe social issue in contemporary China. In evidence, massive rural-to-urban migration has contributed to rising income inequality [23]. On the one hand, most migrant workers are typically less educated than the local population, and massive inflow of uneducated migrants into cities increases the supply of unskilled workers in the local labor market. Due to either skill complementarity or externality, skilled workers would naturally get a higher return from skill premium [24,25,26]. On the other hand, because of household registration and some other restrictions, migrant workers usually get paid less along with lower human capital return than their local counterparts in the labor market [27,28,29,30]. Compared with the non-migrating rural residents, the migrant population experiences substantial upward income mobility. Unfortunately, while millions of migrant workers have made an enormous contribution to the lasting economic growth of host cities, migrants are regarded as second-class citizens suffering severe discrimination. They also encounter various formal and informal obstacles in getting access to a variety of government social welfare [31,32,33]. If local non-farm employment opportunities are available, even at significantly lower wages, many temporary migrants would prefer to stay in their hometown rather than to migrate under current regulation and hardships [34]. Living at the bottom of China’s socio-economic hierarchy, migrants may be more averse to inequality because they belong to the disadvantaged and marginalized group. However, no study has been undertaken linking migrants’ SWB with urban inequality.

Given the low-income level of migrants, rising inequality may give rise to psychological imbalance and higher poverty rates, which further breed crimes and threatens social stability. Using data from the Dynamic Monitoring Survey of Migrant Population in urban China, this paper examines both the individual-level and aggregate-level determinants of migrants’ SWB in host cities. It focuses particularly on how and to what extent the migrants’ income and the urban income inequality affect their SWB. Since Chinese migrants do not form a homogenous group, this paper further explores the SWB heterogeneity based on the migrants’ personal, socio-economic, and regional dimensions. Finally, we attempt to investigate the potential channels through which income and inequality affect SWB. 

This study aims at contributing to the extant literature in the following respects. First, by using the nationally representative dataset from a recent and large-scale migrant population survey in China, it provides a unique assessment of Chinese migrants’ SWB. Second, unlike most previous studies primarily focusing on individual and household socio-economic characteristics, we consider inequality as one of the most important social environment factors affecting SWB. To the best of our knowledge, this is the first research to present the impact of overall income inequality on migrants’ SWB in urban destination areas. Third, we investigate heterogeneity based on the migrants’ *hukou* status, region, and income. Lastly, this paper provides possible explanations for our findings under the background of China, including social comparison concerns, perceived social mobility, and other psychological mechanisms. 

The remainder of this paper proceeds as follows: Section 2 introduces the theoretical framework and reviews related studies on the subject matter. Section 3 lays out the empirical strategy and describes the data. Section 4 presents the results. Section 5 discusses a variety of essential interpretation issues. Finally, Section 6 concludes the paper with some policy implications.

## 2. Theoretical Framework

Our analysis is closely related to the research on the determinants of SWB, which is a highly complex field and one that has been thoroughly explored. Much of the existing work has focused mainly on individual attributes (e.g., age, gender, race, marriage, unemployment, and education) and aggregate characteristics (e.g., unemployment rate, inflation, GDP, climate, and the natural environment) [35]. Arguably, income is the most widely-studied aspect of all the personal factors. The extensive discussion behind income-to-SWB relation is, to a large extent, inspired by the “Easterlin Paradox”, which states that an upsurge in average income does not necessarily raise average SWB. Easterlin [36,37] documented that while per-capita income rose sharply in the US from 1972 to 1991, the average SWB remained virtually constant or even declined over the same period. The same phenomenon was exhibited in postwar Japan. In recent decades, new and more comprehensive data have allowed researchers to further validate Easterlin’s hypothesis. A number of subsequent studies have attempted to identify the relation between income and SWB; however, no clear consensus has emerged, as scholars found varying results, from weak [38,39,40], positive [41,42,43,44,45], to inverted U-shaped correlations [46,47,48].

The explanations for the relationship between income and SWB are divergent because people have different perspectives. Based on the adaption level theory, humans have the ability to get used to positive changes. As their income increases, people develop a corresponding hedonic adaptation, which implies that additional material goods do not necessarily translate into improvements in SWB [49].

Another cause of divergence between income and SWB can be linked to factors unrelated to income, such as family, health, and leisure time. Factors associated with increasing income can result in the adverse consequences that offset the positive effects of income. For example, higher paying jobs come with added stress and pressure, which would then adversely affect SWB. Guoqiang Tian and Liyan Yang [47] develop a theoretical economic model by considering the factors of income and non-income simultaneously. They suggest that there is a critical income level, which is positively correlated to no-material status. Before people reach the threshold, increasing income has led to the rising of SWB. When income goes beyond the threshold level, the increase in income will result in the decline of SWB. 

Last but not least, explanation for the varying relationship between income and SWB is related to the effects of relative income, which is derived from the social comparison theory in psychology. The effect suggests that while there is a positive correlation between SWB and an individuals’ income, there is a negative relationship between SWB and the income of their peers (relative income). When the income of a reference group increases, people’s unchanged or diminishing relative income leads to a decrease in SWB [40,50].

The SWB is not affected only by individual characteristics, but also by the social environment. Recent studies consider inequality as one of the most important social factors affecting SWB. Understanding income inequality shifts the focus from analyzing the trajectory of the individual’s own income towards investigating the income distribution among the different sectors of society. In general, income inequality influences SWB based on three main arguments:

(1) Egalitarian Preference. People are born with dislike for income inequality and are generally hopeful for an equal society. Thurow [51] claims that income equality itself should be considered as a parameter in the individual’s utility function. According to strict theoretical analysis, Thurow concludes that when income distribution is regarded as a pure public good, people’s preference for income equality will increase together with rising income. Morawetz et al. [52] are the earliest who confirm aversions against income inequality from SWB data. They selected two comparable communities from Israel and found that those rural residents living in communities with high income inequality had lower SWB than their counterparts. Subsequent experiments on income distribution preference have validated people’s aversion of income inequality [53,54,55,56]. 

(2) Social Comparison Concerns. People are not only focused on income inequality in general but are also concerned about their place in the income distribution. According to social comparison theories, when people are confronted with income inequality, they would compare their socio-economic standing with proximate others. For example, consider two groups of people from opposite ends of the income distribution as they experience social comparison. For the poor, they would compare their well-being against better-off referents (otherwise known as upward social comparison) [57], which would result in low levels of SWB due to relative deprivation, jealousy, and self-abasement [58,59,60]. On the flip side, the rich would generally have a downward social comparison, which can increase their SWB through changing self-image [61,62]. In fact, previous studies have also shown that upward and downward social comparisons result in distinct outcomes [63]. 

(3) Perceived Social Mobility. Social comparison puts strong emphasis on the individual’s present situation, while social mobility accentuates the future prospects. Income inequality could be viewed as an economic opportunity. Regarding income inequality as motivating, people will make efforts to improve their income which could lead to a positive effect on their SWB. This is called the *“tunnel effect”* (Hirschman and Rothschild [64] proposed the “tunnel effect” hypothesis. Imagine you are driving in a two-lane tunnel with both lanes headed in the same direction. Traffic is jammed as far as one can see. Suddenly the adjacent lane starts to move. Initially you feel better, even if you are still stuck because this signals that the jam has ended and your own lane will start moving soon. But after waiting at a standstill and watching the other lane move for some time, your feelings change: you become envious and furious. You and others stuck in the lane begin to suspect foul play. You begin to search for a way to address the injustice of the situation by drastic action—including making illegal moves, such as crossing the double line that forbids moving from one lane to the other). Alesina et al. [65] find that income inequality significantly decreases the individuals’ SWB in European countries, but has no clear effect on people in the United States. They argue that the pivotal difference is how Americans perceive their country to be a more mobile society than Europe. Americans believe that while increasing their wealth is not easy, there are a number of opportunities available for them to climb the income ladder. Conversely, Europeans have a more pessimistic view on achieving substantial income progress. Milanovic et al. [66] describe people from transition economies to be more tolerant of income inequality because such an imbalance brings opportunities for more income and takes them out of poverty more quickly. Grosfeld and Senik [67] examine the cross-sectional data of Poland from 1992 to 2005, which is the transition time for Poland, and find a turning point between income inequality and SWB. There is a positive correlation between income inequality and SWB before 1996, which then becomes negative. At the early stage of the transition period, people viewed income equality as an opportunity. However, when they later found that it was impossible for everyone to benefit from income inequality, it had a negative effect on their SWB. The authors defend their arguments by pointing out that the year break (1996/1997) is in accordance with rising distrust in the political system and the elite, which could explain the change in taste for inequality. Knight et al. [68] find that at the county level, China demonstrates a positive correlation between the SWB of its rural residents and income inequality, resulting from people’s optimistic prospect of more opportunities for economic benefits. The authors argue that income inequality within a county may be less likely to make people feel relatively deprived—their orbits of social comparison are considerably narrow for that. On the contrary, higher income inequality may indicate greater diversification in the county, which in turn sends a signal of possibilities for economic advancement. The demonstration effect could happen at the individual level (e.g., by providing employment opportunities) or at the village level (e.g., people believe in the potential development of their own village). Therefore, higher income inequality could make people happier. Likewise, Jiang et al. [69] conduct similar studies on China’s urban residents and draw identical results.

## 3. Empirical Strategy and Data 

### 3.1. Empirical Strategy

Our general strategy is to investigate the relationships of income and inequality with the SWB of migrants. In keeping with existing studies in the literature [50,65], we assume the SWB as an ordered categorical variable and run an ordered logit model. We estimate the SWB equation in the following form:

(1) $$SWE_{is}^{*}=\alpha Income_{is}+\beta Inequality_{s}+\gamma {Χ}_{is}+\eta_{s}+\varepsilon_{is}$$

where $SWE_{is}^{*}$ represents the subjective happiness score of migrant i living in s province which s stands for a destination province. Explanatory variables $Income_{is}$ refers to the absolute income of migrant i living in s province. $Income_{is}$ is measured at the logarithm of respondent’s monthly household income (it is worth noting that our estimate result will not be biased measuring the income at the household level instead of individual income due to accessibility of data. Using household income as an indicator measuring the absolute income is commonly applied [50,65,70]). $Inequality_{s}$ indicates income inequality level of s province, and we calculate the Gini coefficient as the main measure of overall income inequality. The set $\;{Χ}_{is}$ is a vector of personal characteristics (i.e., gender, age, marital status, education background, employment status, ethnicity, and migration characteristics) which have previously been found to affect individual SWB. A detailed summary is provided in Table 1. We also add a dummy variable for the cross-sectional province of destination $\left(\eta_{s}\right)$, and a random error term $(\varepsilon_{is})(i.i.d.$).

Estimation of regression Equation (1) is constrained by an unobservable latent continuous variable $SWE_{is}^{*}.$ The observable are three discrete outcomes resulting from the responses to the happiness question (see Section 3.2 for more details), and levels of SWB are given cardinal values assigned to qualitative assessments as follows: 1 = Unhappy, 2 = So-so, 3 = Happy, respectively. An ordered logit model assumes that:

(2) $$\begin{array}{l} \;{\mathrm{SWE}}_{\mathrm{is}}=1,\;{\mathrm{if}\;\mathrm{SWE}}_{\mathrm{is}}^{*}<C_{1}\\ SWE_{is}=2,\;\;\mathrm{if}\;C_{1}\leq SWE_{is}^{*}<C_{2}\\ SWE_{is}=3,\;\;\mathrm{if}\;C_{2}\leq SWE_{is}^{*} \end{array}$$

where $C_{1},\;\;C_{2},\;\mathrm{and}\;C_{3}$ are threshold values. For example, when $\;SWE_{is}^{*}$ is lower than the threshold value $C_{1}$, the respondent feels unhappy. Based on the likelihood function for each response type, a consistent estimator of interest parameters can be approximated by maximum likelihood method under exogenous conditions.

### 3.2. Data 

Our data comes from “the China Migrants Dynamic Survey in 2011 (CMDS 2011)” collected by the National Population and Family Planning Commission, which is a single cross-section dataset. The survey covered all 31 provinces of China, 326 cities and 5850 communities or villages, and the samples were chosen randomly. The probability proportional to size (PPS) sampling technique was used for this survey. Survey respondents are migrants (not hold local household registration) with age ranging from 16 to 59 and have been living in host cities for at least a month. The survey questionnaire collected basic information including employment status, living conditions, public services, social participation, and social integration. The total sampling size is 128,000. 

Because SWB is affected by numerous factors including economic status, occupation, psychology, interpersonal relationship, and self-achievement, it is difficult to precisely measure SWB. In this study, the discussion of SWB is built on the individuals’ response to happy questions. The questionnaire on migrants in the 2011 CMDS includes a variety of questions about migrants’ social participation and subjective perception. The question on happiness to the respondents is: “How happy do you feel in this city?”. Options for answers include “Happy”, “So-so”, “Unhappy” and “It is hard to see”. We excluded the response “It is hard to say” to remove the ambiguity in the analysis (as a robustness check, we also run the regression with putting these answers together with “Unhappy”. The estimation results (reported in the Table A1) remain remarkably stable). The responses were then converted to numerical scores as previously mentioned. This technique of SWB measurement is simple and straightforward, while its validity and rationality have already been widely recognized in psychology [71]. In related economic literature, SWB is regarded as a proxy indicator for personal utility [72], which provides the basis for the reliability of results.

Table 1 lists the main variables used in the estimate function and provides brief definitions and descriptive statistics. In Table 1, we can see that the SWB of migrants is between the level of “Happy” and “So-so”. In the complete dataset, males outnumber female respondents, accounting for 53.1 percent. The average age of migrants is 33, and the proportion of unmarried samples is 20 percent. Ninety-three percent of those sampled are of Han ethnicity, while 85% originates from rural communities. Inter-province migrants make up half of the samples. As for educational background, about 72% do not have a high school diploma, and only 8% have received college education. Most of the respondents do not possess pension insurance (85%), and more than half come from communities where they live in poor economic conditions (52%). Unemployment among the respondents is seldom (1%). The vast majority of those asked are willing to integrate into their host cities (93%).

Table 2 shows a summary of reported SWB values of migrants. The overall percentage of respondents with “Happy” and “So-so” are 39.28% and 56.48% respectively, indicating that the level of satisfaction among migrants in China is above the halfway mark. When the samples are regrouped by household registration status, region, migration range, and income level, similar results are obtained. Interesting, among those who reported “Happy”, the percentage of SWB is higher for rural migrants than for their urban counterparts (2.06% more); similarly, the reported “Unhappiness” is lower for rural than urban migrants (1.48% less). Income seems to be an important factor in influencing the migrants’ SWB. The percentage of individuals reporting “Happy” is substantially lower for those in the lowest-income group than for those in the middle- and highest-income groups.

## 4. Results 

### 4.1. Baseline Results

Table 3 presents the results of the estimation of the ordered logit model utilizing the complete dataset of migrants. In column 1, only the control variables are included in the regression equation. Generally, all the estimates reflecting the respondents’ personal attributes are statistically significant. These sensible results provide some confidence in the structure of the responses to the SWB question. The effect of age is estimated to be nonlinear. Migrants, aged 25 to 35, seem to have the least SWB levels, which could be traced from the enormous professional and personal pressure faced by this particular age group. Women are happier than men, probably because men usually carry more economic pressures and social responsibilities in Chinese society. Rural migrants are shown to be significantly happier than urban migrants, despite commonly being more at a disadvantage economically. This could be explained by the group’s inherent differences in terms of aspirations, income expectations, and assessment for improvement of life [73]. Belong to ethnic minorities is associated with higher levels of SWB, maybe because they have a positive lifestyle and strong inner capacity for pursuing a happy life [74]. Married individuals are happier than unmarried ones possibly due to the fulfillment they receive from family life.

Inter-province migration brings disutility, most likely because of the long distance to hometown. The longer migrants stayed in host cities, the happier they are. Unemployment, lack of pension insurance, and economic stresses tend to decrease the SWB levels. Social inclusion is found to have significantly positive relationship with migants’SWB. Social inclusion provides the migrants the means to gradually integrate, to assimilate with the surrounding environment, and to develop self-adjustment and psychological identification. Migrants will feel valued and important within an inclusive city where they can actively participate and achieve their full potential. These findings are consistent with previous studies related to SWB [45,50,68]. Surprisingly, more educated migrants report lower SWB. Education is commonly considered as providing more opportunities for higher income, which would then lead to increased SWB; however, this is contrary to the results found in this study. One possible explanation could be that people generally create higher expectations for their well-being as they become more educated [75]. Migrants with higher educational background expect more from life and become more reluctant to accept poor living circumstances. 

Focusing on understanding the relationships of income and inequality with SWB, the variable absolute income is added in the model (column 2). The coefficient is 0.199, different from zero at a significance level of 1%. This indicates that rising income categorically results in higher SWB among Chinese migrants. To simply compare effects of absolute income and income inequality on SWB across columns, we added a bold number below for standard error of each coefficient: these numbers are the effect on the probability of moving from one level of SWB to the next, as a result of one standard deviation change in the absolute income or income inequality (in extremely large samples, it may reduce the credibility of results to solely rely on the sign of coefficient and low p-values to support claims [76]. In this regard, we have reported the effect sizes of explanatory variables and their 95% confidence intervals). Specifically, implications of these numbers are as follows: when it is a negative (positive) number, it refers to the proportion of people who leave (enter) the top SWB category “Happy” and consequently enter (leave) one of the bottom two SWB categories, ‘‘So-so’’ or ‘‘Unhappy’’. (for ordered logit, the conversion from coefficients to probability effects is calculated using the formula: ∆Probability (Person being in top SWB category) =1−1/(1+exp(score+coefficient×∆X−_b[_cut2])) −(1−1/(1+exp(score−_b[_cut2]))), where ∆X is the change in the explanatory variable, _b[_cut2] is the top cut point and score refers to the predicted value of the underling continuous variable. This approach also appears in Alesina et al. [65]. For further discussion of the partial effects of the coefficients in ordered choice models, please see Greene and Hensher ([77], chap. 5.) A rise in the absolute income of one unit leads to an increase in the proportion of people reporting themselves as “Happy” of 4.6% and a fall in the proportion reporting themselves as “Unhappy” of 0.7%. Since 39.28% of migrants reported themselves as “Happy”, an increase in absolute income of one unit is expected to bring about an 11.7% increase in the number of migrants reporting themselves in the top SWB category (=4.6/39.28). Since 2.24% of migrants reported themselves as “Unhappy”, it would also correspond to a 16.5% reduction in the number of migrants reporting themselves in the bottom SWB category.

Income inequality variable is added in column 3. In this model, absolute income is still shown to be a statistically significant predictor for SWB and with a positive coefficient. However, the coefficient on income inequality is negative and significant at 1% level, even after we control for absolute income. In other words, income inequality is detrimental to the SWB of migrants. A 10% increase in inequality reduces the proportion of people reporting themselves as “Happy” by 7% and increases the proportion reporting themselves as “Unhappy” by 1.5%. These figures would also correspond to a 17.8% reduction in the number of migrants self-declaring themselves as being “Happy” (from 39.3% to 32.3%) and an increase of 35.4% of migrants self-declaring themselves as being “Unhappy” (from 4.8% to 6.5%). These effects are quite large. In this study, having a negative correlation of income inequality with SWB does not support the idea of a positive tunnel effect. 

The square of the absolute income variable is added to test the hypothesis that further income contributes less to well-being as income rises (presented in column 4). The coefficient of the squared term is significantly negative, indicating an inverted U-shaped relationship between absolute income and SWB. When income goes beyond a certain threshold (monthly household income of about 20,000 *yuan*), higher income will not result in more gains in SWB. The result supports the phenomenon of “Easterlin Paradox”. Meanwhile, the sign and significance of the coefficient for inequality are unaffected.

### 4.2. Heterogeneity Analysis

#### 4.2.1. The Heterogeneous Effects of Household Registration Status and Region 

Numerous studies have shown that household registration status can substantially influence the SWB for both urban and rural residents [27]. After China joined the WTO in 2001, migrants, mostly coming from rural areas, flowed into the cities in massive numbers. Because of the impediments created by the household registration rules, a three-strata economic structure society, organized with Chinese characteristics, has been established. This society incorporates rural residents, city inhabitants, and migrant workers. The relationship between SWB and household registration status of migrants remains unclear. In the meantime, economic development continues to be unbalanced among the different regions in China for a long time. After investigating the whole sample, we further explore the SWB heterogeneity based on household registration status (rural vs. urban) and geographic locations (eastern, central, and western regions). We run separate estimations to identify differences in SWB among different groups. 

From the results, as demonstrated in columns 1 and 2 of Table 4, absolute income is shown to have significant positive effects on both the rural and urban migrants. We then analyze the incremental effects on SWB of modifying explanatory variables. For rural migrants, if the absolute income increases by one unit, it leads to an increase of 18.4% in the number of migrants self-declaring themselves as being “Happy” and a decrease of 20.9% of migrants self-declaring themselves as being “Unhappy”. As for urban migrants, the effects correspond to the number of migrants reporting as “Happy” increases by 12.5%, while the “Unhappy” migrants decrease by 15.1%. As shown by the results, the effect of income in the subsample of rural migrants is larger than the effect of income in the subsample of urban migrants. This result is in accordance with the general finding in the literature, which suggests that absolute income is relatively more important for individuals with lower income than for higher income ones (i.e., rural migrant workers have relatively lower income than urban migrants). In columns 1 and 2, the quadratic coefficients of absolute income are negative, indicating that the relationship between income and SWB of migrants (both rural and urban) is an inverted U-shaped pattern. Also, income inequality is shown to negatively affect the SWB for both rural and urban migrants, but we find that the impact of income inequality on SWB is not significantly different between *hukou* status.

Columns 3, 4 and 5 of Table 4 show the regression analysis for migrants living in eastern, central, and western regions, respectively. An inverted U-shaped relationship between absolute income and SWB exists in all three regions. Further calculations suggest that if absolute income increases by one unit, it would generate an 11.8% increase in “Happy” and a 16.6% decrease in “Unhappy” among migrants in the eastern region. For the migrants in central and western regions, the rates of reporting “Happy” would increase by 21.5% and 24.8% and would decrease “Unhappy” migrants by 24.3% and 27.9%, respectively. Due to gaps in regional development, migrants living in central and western regions have lower income than their eastern counterparts. This reaffirms the observation that income effect is greater in lower income groups. 

Additionally, income inequality has a significant negative effect on migrants in all regions. We find surprising results after evaluating the model coefficients. The negative effect brought by income inequality on migrants in the western region is much higher than migrants in the central and eastern regions. According to the result, when income inequality increases by 10%, the rate of “Happy” in the western region decreases 30.3%, the reported “Unhappy” rate increases by 69.9%. The figures in the central and eastern regions show similar decline in “Happy” rates (16.6% and 30.6%) and rise in “Unhappy” rates (16.5% and 31.8%). Once again, these negative effects are quite large.

The results of the regression models (Table 4) are then compared with the regression model (4) in Table 3. There is no substantial difference in terms of sign, significance, and size of coefficients. This indicates that the selected control variables can provide adequate explanations as to what affects perceived well-being. We report again the coefficients of control variables, as there are some interesting differences across groups. We find that gender difference has little effect on the SWB of rural workers. Unemployment has a significant negative effect, but only for rural workers. One possible justification is that because urban workers are provided with more social welfare. Inter-city (within a province) and inter-county (within a city) migration have adverse effects on migrants’ happiness in the eastern region. We argue that this is directly related to the region’s migrant demographics. The inter-province migration in the eastern region is comprised mainly of migrants from the central and western regions. Migrants from within the province and those coming from other provinces are distinctively divergent with respect to economic conditions and expectations. Under social comparison, when the reference object is different while other conditions remain unchanged, perceived well-being tends to be lower. The impact of these variables on SWB can be compared with the effect of income inequality. Let us take a rural worker as an example: if we increase one standard deviation of income inequality, its negative effect is equal to 81% of the impact of decreasing one standard deviation on duration in this city (this number equals 81%=0.039×3.545/ (0.034×5), where 0.039 and 5 are the standard deviations of income inequality and duration in this city, respectively, taken from Table 1; 3.545 and 0.034 are the absolute values of the coefficients of income inequality and duration in this city, taken from column 1 of Table 4). It is also equivalent to 2.8 times the effect of having no pension insurance, or 70% impact from unemployment (these numbers equal 2.8=0.039×3.545/0.050, 70%=0.039×3.545/0.198, respectively, where 0.039 is the standard deviation of income inequality, taken from Table 1; 3.545, 0.050 and 0.198 are the absolute values of the coefficients of income inequality, pension insurance and unemployed, taken from column 1 of Table 4).

#### 4.2.2. Heterogeneity across Income Groups 

In Table 5, absolute income is divided into quartiles to further examine the effects of income and inequality across income groups. For this purpose, migrants are separated into three sub-samples: “lowest”, “highest” and “middle”. A respondent is classified as ‘‘lowest’’ if he/she is in the bottom income quartile, as ‘‘highest’’ if he/she is in the top income quartile and as “middle” otherwise. As reported in Table 5, absolute income has significant positive impact on the happiness of migrants among middle-income and lowest-income groups, but it is not a significant indicator for the highest-income group. Again, this highlights a turning point in absolute income and how it impacts SWB. There is satiation point beyond which additional income would not necessarily increase perceived well-being.

As shown in Table 5, income inequality has detrimental impact on all three income groups, which are statistically significant at 1% level. However, the effect of inequality significantly differs among the three classes. As indicated by the inequality coefficient, the value is about three times bigger in the highest-income group than the lowest-income group. If income inequality is increased by 10% in the lowest-income group, it will lead to a 9.9% decrease in satisfied migrants and a 15.9% increase in dissatisfied respondents. For the highest-income group, the change would be 23.5% decrease in satisfaction and 53.9% increase in dissatisfaction. 

### 4.3. Robustness Checks

In what follows, we perform a series of checks to show that our results are very robust. The relevant results based on alternative estimates are presented in Table 6.

First, even though a large set of individual characteristics are controlled in the analysis of migrants’ SWB, some individual unobservable variables (such as personality traits) may have been omitted. Considering that SWB depends on the individuals’ subjective attitudes, we add another two control variables (namely how migrants think about host cities and local people) to alleviate the concern of the potential omitted variable bias. As the Gini coefficient is a relative measure of inequality, it cannot reflect absolute income gap among people’s incomes. Using absolute rather than relative measure of income inequality may give us a very different picture of inequality [78]. In the second check, we replace the core explanatory variable, using the Absolute Gini index to measure income inequality. Third, Ferrer-i-Carbonell and Frijters [79] argue that if the model is correctly set-up, the sign and significance of the interest coefficients are robust for either OLS or ordered logit regression models. In estimating the SWB model, we have used the ordered logit regression. As a check, an OLS estimate is ran to analyze SWB in Chinese migrants. Last, in order to overcome the contingency of one-year data, “the China Migrants Dynamic Survey in 2012 (CMDS 2012)” is used for robustness check. The sampling design of the CMDS 2012, based on the sampling scheme of the CMDS 2011, has improved in respect to the overall sample size, sample allocation and sample frame. The survey is nationally representative of China, with the respondents randomly selected from each of the 1171 counties employing stratified, multistage clustered, and Probability Proportionate to Size (PPS) sampling. The questionnaire used in the CMDS 2012 is basically the same with the CMDS 2011, but with some of the questions improved. For example, a 5-point scale has been applied to measure SWB. Unfortunately, the CMDS 2012 is still a single cross-section dataset, which means that the respondents in 2012 will be different migrants to those in a prior year. We could only partially test the robustness of our results given that we lack panel information on SWB over time.

As shown in Table 6, all robustness test results are consistent with the primary results above in terms of both the signs and significance of the estimated coefficients. These checks provide great support for the reliability and validity of our main findings. 

### 4.4. Accounting for Endogeneity of Income

Thus far, we have assumed that the error term $\varepsilon_{is}$ in Equation (1) is independent of income. This strong assumption may lead to endogeneity problems when unobserved confounding variables are not adequately considered. For example, there might be a problem of reverse causality: happy migrants may make more money because they are more optimistic. However, given the wide variety of factors that may influence happiness, it is difficult to find an instrumental variable directly linked with income but indirectly related to happiness. It is noteworthy that this endogeneity problem is a challenge to be overcome in the field. We implement an alternative approach proposed by Lewbel that exploits heteroskedasticity for identification in the absence of traditional identifying sources, such as external instruments [80]. In particular, the identification can be achieved by having regressors uncorrelated with the product of heteroscedastic errors in the first stage regression. As shown by Lewbel, proper instruments can be constructed from the first-stage regression’ residuals, multiplied by each (or a subset) of the included exogenous regressors in mean-centered form: $Z_{j}=\left(X_{j}-\bar{X}\right)∆\varepsilon$. We use Bresusch-Pagan test for the presence of heteroskedasticity. In our case, the test result clearly rejects the null hypothesis of homoskedastic errors with a p-value equal to 0.00. In the next step, we use the set of generated instruments to re-estimate the results reported in column 4 of Table 6. Our model is estimated with both the two-stage least squares (TSLS) and generalized method of moments (GMM) methods. Table 7 shows that our results are qualitatively unchanged using the alternative identification scheme due to Lewbel. The GMM estimation technique provides an asymptotic efficiency gain over the standard TSLS estimator. The usual diagnostic tests give us supportive evidence on the validity of the instruments. 

## 5. Discussion

### 5.1. Interpreting the Effects of Income and Inequality

While an inverted U-shaped pattern is shown to generally describe the relationship between SWB and income, the positive effect of income is more prominent on the SWB of rural workers and those living in Western China. For migrants in the highest-income group, increasing income levels does not necessarily translate into better perceived well-being. In order to explain our findings, we further submit the following rationalizations: 

(1) For most people, income serves as an economic base in order to meet basic needs and increase the SWB [81,82,83]. At present, China is in the stage of development with the enormous scale of rural-to-urban workers. In this situation, rising income levels can bring improvements in the quality of life and economic security for the individual and his or her family. Particularly those migrants living in bad economic conditions, income is an essential component of improving well-being. However, component of well-being comprises not just finances but other things such as family, work, health and leisure [84]. Once basic physical needs have been largely met, higher income might no longer bring about greater well-being. 

(2) Social comparison is a basic feature of human social life [85]. When an individual’s income increases relative to one’s reference group, such an increase commonly results in increased SWB. However, if the person’s income rises alongside the general income of one’s reference group, then the increase is probably no longer associated with greater SWB [86,87]. In Table 8, we evaluate the average income for each province and use it as reference value. The relative income is then calculated by getting the difference between individual income and reference value. As presented in column 1 of Table 8, the increase in relative income leads to a significant increase of SWB. 

(3) In general, SWB is positively correlated with income and is negatively correlated with additional material aspirations. When people adjust to higher income levels, they may develop new expectations. Initial pleasure in increased satisfaction from rising income wears out over time, similar to the waterwheel with constant rising to the peak and then descend back to the origin [88,89]. 

(4) In the pursuit of generating higher income, people may engage in activities causing higher amount of tension and stress [90], which could drive decreases in SWB. Meanwhile, time for positive experience (for example, leisure activities) may be reduced [91], which may ultimately negate the positive impacts of income increase.

Income inequality is found to have a negative effect on migrants’ perceived well-being for a variety of possible reasons. The first one involves an individual’s taste for income inequality (i.e., her preferences for equality). Income inequality is generally regarded as a disgusting product– something most people dislike [92]. In China, there may be higher egalitarian preferences. Influenced by Chinese traditional culture and past socialist egalitarianism, the Chinese people are less worried about poverty, but rather are more concerned with the uneven distribution of wealth [93]. The second possible reason we discuss is social comparison. In column 2 of Table 8, we find that the interaction term of income inequality and relative income is positive, suggesting that income inequality has more serious effects on the lowest-income group. Migrants in the lowest socio-economic classes in China experience relative deprivation as a result of social comparison, which may decrease their SWB. However, controlling for absolute income and relative income (as shown in columns 3 and 4 of Table 8), the interaction term of income inequality and relative income becomes negative, which indicates that the social comparison theory does not explain the strong native effect of income inequality on high-income migrants. Another possible explanation we argue is the migrants’ high expectations. When they made decisions to migrate, prospective migrants may not be able to form rational expectations about future urban conditions, or about their future urban aspirations, or about their future selves [10]. After they become part of the new and very different urban society, the gap between their relatively high expectations and their relatively low socio-economic status may reduce their happiness. To test whether high expectations affect the relationship of income inequality and SWB, we use a dummy measure of agreement with the statement “I am concerned with changes in the city” from the CMDS as a proxy for expectations. In addition, we believe that migrants from farther areas may have greater expectations for their future. In this sense, we use migration distance (inter-province migration = 1, intra-province migration = 0) as a supplementary measure of expectations. Table 9 includes the interactions between income inequality and variables that tap into the expectations mechanism. We evaluate the interactions and find significant negative effects, meaning that migrants who have higher expectations experience lower SWB in high income inequality. Finally, we consider the role that perceived fairness has in explaining the effect of income inequality on SWB. In general, individuals do not only care about the outcomes they achieve but also about the fairness of the processes that generated these outcomes [94]. The source of income inequality (e.g., birth, effort, injustice) may affect an individual’s tolerance for income inequality. Chinese migrants are subject to discriminations in access to jobs, medical care, public housing, and children education. City governments favor local residents, and migrants in cities are generally treated as second class citizens. Thus, unfair treatment of migrants in cities may contribute to the negative impact of income inequality on their happiness. We operationalize perceived fairness as migrants’ agreement with the statement “I feel the locals always look down on outsiders”. In column 3 of Table 9, we find support for the hypothesis of perceived fairness. In other words, at high levels of income inequality, migrants have lower SWB if they believe that they have been treated unfairly in cities.

Our empirical results have also shown that income inequality has a greater negative effect on SWB for migrants living in the western area and those with high income. In the following analysis, we discuss the role of social mobility on the heterogeneous effects of income inequality. Migrants in the western area are more concerned with inequality than in the eastern and central regions, possibly because the west relatively lacks social mobility. This claim is consistent with previous studies on the regional structure of the social mobility of Chinese residents [95,96]. In the absence of social mobility, it becomes more difficult for western migrants to move up the income ladder, which makes them feel less optimistic about their economic future and thus have a lower tolerance towards income inequality. 

For the situation of migrants with high income, this could also be plausibly explained from the perspective of social mobility. As a result of China’s rapid urbanization, migrants have experienced substantial upward mobility, and the high-income group of which has become the middle class in Chinese social stratification [97,98]. But compared with middle- or lower-income migrants, upward mobility is much harder for high-income migrants despite their advantages in socio-economic status. There are two main reasons for this point. First, the household registration system, to a large extent still is a difficult hurdle that high-income migrants encounter [99,100]. This institutional barrier restricts them from obtaining more social welfare and local residence rights, such as earnings, medical care, public housing, children education, pension and unemployment benefits. Their efforts probably do not translate in achieving their desired outcomes. Second, like the middle class in China, high-income migrants may undertake a great deal of stress along with intense social competitiveness in a more unequal society [101]. In order to maintain their achieved socio-economic status, they suffer more than their counterparts from heavy workload, soaring housing prices, and expensive costs of raising a family. At the same time, they are also greatly concerned about the threats to property brought about by economic reforms, which could potentially downgrade them into becoming part of the low-income class in the future [102,103]. Therefore, for high-income migrants who perceive the potential for downward mobility in their society, they will view current income inequality as a predictor of future relative poverty and thus would be more adversely affected by inequality [104,105].

Ferrer-i-Carbonell and Ramos surveyed the empirical literature on the relationship between income inequality and happiness [106]. They concluded that income inequality is negatively related to happiness in Western countries, whereas the effect of income inequality on happiness is mixed in non-Western countries. They pointed out that due to fast changing, volatile, and specific situations of those countries in transition, the studies on an individual’s taste for income inequality have not been conclusive. Our results are contrary to another study that has also used Chinese data. Jiang et al. found that overall income inequality positively correlates with happiness using the data from the 2002 Chinese Household Income Project (CHIP) Survey [69]. With different target populations, we consider that our results are not in direct contradiction with their conclusions. The samples of Jiang et al. are urban residents and migrants, of which the urban residents account for nearly 70% (3797/5630). At the same time, the 2002 CHIP collected information on migrant neighborhoods in only five provinces [107], while our dataset covered all 31 provinces of China. Also, they did not distinguish between the two samples when estimating the relationship between overall income inequality and happiness. Understandably, there is a huge difference between the urban population and the migrant population in terms of socio-economic status. Their conclusions may be more applicable to mainly urban residents. The authors provided one possible explanation why they found a positive association between income inequality and happiness. They defend that people may optimistically expect more opportunities for economic advancement in the period of rapidly increasing incomes. We can think of potential alternative explanations, although the authors did not exploit them. Urban residents may perceive the current income inequality to come about through fair processes. In addition, as urban residents generally earn higher incomes than migrants, they may less likely to feel relatively deprived in upward social comparisons. Furthermore, as mentioned in the Introduction, the skill premium has made urban residents the beneficiaries of income inequality, as large numbers of unskilled rural-urban migrants have increased urban income inequality. Conversely, compared with the urban population, less educated and unskilled migrants may find greater difficulty to increase their income. Also, being ranked lower in the city income distribution and suffering from unfair treatment in cities, migrants are more likely to dislike income inequality. All in all, because of these notable differences between migrant versus non-migrant groups, individuals may have heterogeneous preferences over income inequality. 

### 5.2. Limitations and Future Directions 

It is noteworthy that the current study is subject to several notable limitations. First, although we utilized a nationally representative dataset from the CMDS, our study is a cross-sectional design and cannot follow individuals over time. Its cross-sectional nature means that some of our tests are not strong enough, and some claims have been left unexplored. For example, migrants with low SWB and low income may tend to cluster in the provinces with higher inequality. Future work needs to provide more robust evidence on the causal chain using longitudinal dataset and experimental or quasi-experimental designs. Second, constrained by the available data sources, it’s almost impossible for us to make conclusive tests of the hypothesis concerning perceived social mobility among migrants. As both datasets continue to expand and contain more psychological variables, additional studies are encouraged to further shed light on the ways how individual beliefs and attitudes (e.g., subjective social status) drive the differences in the relationship between income inequality and well-being across groups. Third, this study only focused on migrants and did not attempt to provide a comparative analysis between migrants and non-migrants. Future research can compare the happiness among different groups and study how different inequality affects migrants from non-migrants. Fourth, we did not consider the causal impact of migration on the happiness of migrants. Counterfactual analysis is much needed to examine the effects of rural-to-urban migration on Chinese migrants’ well-being and to disentangle the specific channels at work. Lastly, we limited social comparison mainly in terms of income, but comparing SWB in itself may affect people’s happiness [108]. The inequality of SWB has been characterized as an informative indicator of social tensions [109]. The current SWB literature still largely lacks studies on happiness inequality. In this regard, an important area for future studies may seek to explore the links between happiness inequality and individuals’ SWB. Likewise, since happiness is an ordinal variable, the measurement of happiness inequality could also be worth studying [110].

## 6. Conclusions

Understanding the well-being of China’s migrant workers is an extremely important issue not only to protect potentially vulnerable people, but also to provide a safer, happier, and more equal Chinese society. The current understanding concerning the determinants of SWB, particularly how the social environment, such as income inequality, affects Chinese migrants’ SWB remains unknown. In this paper, we examine the relationship between absolute income, income inequality, and SWB, using data from an extremely large and nationally representative migrant population survey in China. 

The main findings can be summarized as follows: (1) Absolute income is an economic base for migrants to improve their SWB. Absolute income is shown to have a positive effect on migrants’ SWB, and their relationship can be illustrated as an inversed U-shaped pattern. Since migrants generally tend to have low income, the vast majority of migrants have not reached the income level at “satiation point of SWB”; (2) Income inequality has serious negative impacts on SWB. And the findings in this study do not support the idea of a positive tunnel effect; (3) There are heterogeneous income effects when comparing the migrants’ SWB by *hukou* status, region, and income. For rural workers with relatively lower income and migrants from the central and western regions, absolute income has greater positive impacts on SWB. In contrast, increase in absolute income substantially brings more happiness in middle- and low-income migrants, compared with their high-income counterparts; (4) For migrants with different household registration, there is no significant difference on the negative impact of income inequality on their SWB. However, for migrants living from different regions and from different income groups, the negative impacts of income inequality on SWB show clear heterogeneity. Moreover, income inequality has greater negative impact on migrants in the western region and migrants with high income. (5) The results of the control variables appear to be sensible and are consistent with the SWB literature (i.e., age has a nonlinear effect with SWB).

Although millions of migrant workers have made enormous contributions to the lasting economic growth of host cities, they belong to the disadvantaged and marginalized group. An important goal for the Chinese government is to help migrants integrate into local cities. The findings of this research contain several policy implications: (1) Government can be more attentive and provide more support for migrants to increase their income in various aspect. First, major contributory factors for migrants’ low income are the discrimination and restrictions in the urban labor market (e.g., *hukou* discrimination, and exclusion of migrants to specific jobs). The government should provide equal employment opportunities for migrants and eliminate the employment policies that restrict them. Second, the government should improve the employment service system in host cities. Public employment service agencies in cities should be open to migrants and provide free employment information and services. Finally, the government should try to improve the competency and proficiency particularly among the migrant population. While increasing investment in rural basic education and vocational education, the government should pay special attention to employment training for migrant workers. (2) The income distribution system can be adjusted and increase the wages of migrant workers. The government can raise the minimum wage standard to ensure that low-income workers get reasonable labor remuneration and provide certain tax incentives for migrants through the personal income tax. In the redistribution agenda, the government should prioritize support of the middle- and low-income groups. Due to the restrictions of household registration system, migrants are largely excluded from the urban welfare system and do not have full access to local public services and social welfare programs. The government should further break the institutional barriers such as household registration system and promote equality in basic public services. Moreover, more stable transfer payment system of incentive compatibility should be established to narrow the income gap between economic classes and realize harmonious regional development. The income gap between urban and rural areas and the disproportionate regional development are the primary agents of population mobility. The government should promote equitable economic development and public services in urban and rural areas and in different regions, to alleviate the problems associated with large-scale migration. (3) The government must promote greater economic mobility by providing more opportunities for income growth in the workforce. Compared with local workers, migrants are usually at a disadvantage particularly in terms of job options, income level, and social welfare. The government should strive to eliminate the "market access qualification" to guarantee equal access to market resources. Efforts should be undertaken to break monopolies in the market and allow fair competition to improve efficiency in resource allocation and promote equal opportunities for all.

## Figures and Tables

**Table 1:** Variable Definitions and Descriptions.

Variable	Definitions	Obs.	Mean	S.D.	Min	Max
SWB	Unhappy = 1, So-so = 2, Happy = 3	116,134	2.350	0.559	1	3
Income	Log household monthly income (*yuan*)	115,655	8.123	0.560	5.3	11.9
Inequality	Province-level Gini coefficient	32	0.350	0.039	0.29	0.43
Male	Male = 1	116,134	0.531	0.499	0	1
Age		116,134	33.514	9.173	16	59
Rural *hukou* status	Rural *hukou* status = 1	116,064	0.850	0.357	0	1
Han ethnicity	Han ethnicity = 1	116,134	0.931	0.254	0	1
Junior high school or lower	Junior high school or lower = 1	116,134	0.719	0.449	0	1
High school	High school = 1	116,134	0.204	0.403	0	1
College or higher	College or higher = 1	116,134	0.077	0.226	0	1
Inter-province migration	Inter-province migration = 1	58,325	0.503	0.500	0	1
Inter-city in a province	Inter-city in a province = 1	36,377	0.313	0.464	0	1
Inter-county in a city	Inter-county in a city = 1	21,351	0.184	0.387	0	1
Unmarried	Unmarried = 1	116,134	0.204	0.403	0	1
Unemployed	Unemployed = 1	116,134	0.014	0.115	0	1
Duration in this city (years)		116,134	4.724	5.012	0.1	58.9
Pension insurance	Urban pension insurance coverage = 1	114,588	0.151	0.358	0	1
Economic strain	With economic strain = 1	116,134	0.516	0.546	0	1
Social inclusion	Willing to integrate into this city = 1	116,134	0.93	0.255	0	1

Data sources: The 2011 China Migrants Dynamic Survey and authors’ calculation.

**Table 2:** SWB of migrants in China.

**Reported SWB**	**All**	***Hukou* Status**		**Region**		
		**Rural**	**Urban**	**Eastern**	**Central**	**Western**
Happy	39.28	39.60	37.54	38.18	40.02	39.95
So-so	56.48	56.38	56.96	56.66	56.40	56.33
Unhappy	4.24	4.02	5.50	5.17	3.58	3.72
**Reported SWB**	**Migration Distance**			**Income Groups**		
	**Inter-Province**	**Inter-City in a Province**	**Inter-County in a City**	**Lowest**	**Middle**	**Highest**
Happy	37.48	41.25	40.78	33.38	39.84	43.89
So-so	57.71	54.95	55.74	61.58	56.34	51.84
Unhappy	4.80	3.80	3.47	5.04	3.82	4.27

Data sources: The 2011 China Migrants Dynamic Survey and authors’ calculation. Note: All numbers are expressed as a percentage. A respondent is classified as ‘‘lowest’’ if he/she belongs to the bottom income quartile, as ‘‘highest’’ if he/she belongs to the top income quartile and as “middle” otherwise.

**Table 3:** Determinants of Migrants’ SWB: Full Sample.

Dep Var: SWB	(1)	(2)	(3)	(4)
Income		0.199 *** (0.032) **0.0258** **[0.0230, 0.0286]**	0.209 *** (0.029) **0.0271** **[0.0243, 0.0299]**	1.311 *** (0.252) **0.0310** **[0.0286, 0.0334]**
Inequality			−3.203 *** (0.211) −**0.0282** **[**−**0.0255**, −**0.0308]**	−3.110 *** (0.211) −**0.0274** **[**−**0.0247,**−**0.0301]**
Income squared				−0.066 *** (0.015)
Age	−0.028 *** (0.007)	−0.032 *** (0.007)	−0.029 *** (0.007)	0.031 *** (0.007)
Age squared/100	0.044 *** (0.010)	0.050 *** (0.010)	0.048 *** (0.010)	0.050 *** (0.010)
Male	−0.042 ** (0.017)	−0.048 *** (0.017)	−0.048 *** (0.017)	−0.051 *** (0.017)
Rural *hukou* status	0.109 *** (0.034)	0.116 *** (0.034)	0.116 *** (0.034)	0.113 *** (0.033)
Han ethnicity	−0.259 *** (0.068)	−0.284 *** (0.069)	−0.321 *** (0.061)	−0.328 *** (0.061)
Unmarried	−0.127 *** (0.038)	−0.034 (0.037)	−0.028 (0.035)	−0.010 (0.034)
Inter-provincial migration (Reference group)				
Inter-city in a province	0.147 ** (0.071)	0.170 ** (0.071)	0.170 ** (0.070)	0.171 ** (0.070)
Inter-county in a city	0.143 ** (0.064)	0.174 *** (0.065)	0.167 *** (0.060)	0.169 *** (0.060)
Duration in this city	0.036 ** (0.003)	0.034 *** (0.003)	0.034 *** (0.003)	0.034 *** (0.003)
Junior high school or lower (Reference group)				
High school	−0.147 *** (0.027)	−0.165 *** (0.026)	−0.152 *** (0.024)	−0.151 *** (0.024)
College and higher	−0.257 *** (0.037)	−0.304 *** (0.040)	−0.288 *** (0.038)	−0.286 *** (0.037)
Pension insurance	0.094 * (0.057)	0.079 (0.058)	0.067 (0.054)	0.064 (0.054)
Economic strain	−0.304 *** (0.028)	−0.264 *** (0.029)	−0.253 *** (0.028)	0.252 *** (0.028)
Unemployed	−0.210 *** (0.063)	−0.133 ** (0.066)	−0.144 ** (0.065)	−0.137 ** (0.065)
Social inclusion	1.133 *** (0.048)	1.131 *** (0.048)	1.129 *** (0.047)	1.131 *** (0.047)
Obs.	114,440	113,995	113,995	113,995
Log-likelihood	−91,938.3	−91,392.2	−91,181.7	−91,150.4
Pseudo-R^2^	0.025	0.026	0.029	0.029

Notes: All regressions also contain province dummy variables, not reported here for brevity. City-clustered robust standard errors are in parentheses for ordered logit regressions. * *p* < 0.10, ** *p* < 0.05, *** *p* < 0.01 (two-tailed z tests). The cell in bold below the standard error of the Income and Inequality coefficient reports the predicted change in the proportion of people in the bottom or top SWB category due to a one standard deviation change in the corresponding explanatory variable (see text for more details). The numbers in the brackets are 95% confidence intervals of effects sizes (please refer to the text for detailed information).

**Table 4:** Heterogeneous effects by household registration status and region.

Dep Var: SWB	Rural	Urban	Eastern	Central	Western
	(1)	(2)	(3)	(4)	(5)
Income	1.395 *** (0.294) **0.0431** **[0.0368, 0.0494]**	0.792 ** (0.387) **0.0309** **[0.0271, 0.0347]**	0.748 ** (0.323) **0.0262** **[0.0206, 0.0318]**	2.568 *** (0.451) **0.0503** **[0.0423, 0.0584]**	2.936 *** (0.384) **0.0617** **[0.0583, 0.0651]**
Income squared	−0.071 *** (0.017)	−0.036 * (0.021)	−0.034 * (0.019)	−0.136 *** (0.027)	−0.044 *** (0.023)
Inequality	−3.545 *** (0.266) −**0.0291** **[**−**0.032,**−**0.0262]**	−3.294 *** (0.295) −**0.0344** **[**−**0.0413,**−**0.0275]**	−2.894 *** (0.365) −**0.027** **[**−**0.0314** −**0.0226]**	−2.959 *** (0.196) −**0.0253** **[**−**0.0306,**−**0.0199]**	−5.740 *** (0.863) −**0.0431** **[**−**0.0476,**−**0.0385]**
Age	−0.029 *** (0.008)	−0.041 *** (0.012)	−0.013 (0.010)	−0.046 *** (0.015)	−0.038 *** (0.011)
Age squared/100	0.049 *** (0.011)	0.056 *** (0.016)	0.029 ** (0.014)	0.073 *** (0.021)	0.054 *** (0.014)
Male	−0.044 *** (0.017)	−0.083 ** (0.040)	−0.052 * (0.028)	−0.048 (0.034)	−0.060 *** (0.020)
Rural *hukou* status			0.143 *** (0.038)	−0.006 (0.078)	0.235 *** (0.051)
Han ethnicity	−0.365 *** (0.067)	−0.141 ** (0.070)	−0.139 * (0.075)	−0.198 ** (0.088)	−0.302 *** (0.080)
Unmarried	0.022 (0.037)	−0.150 ** (0.067)	−0.040 (0.050)	0.092 (0.083)	−0.041 (0.049)
Inter-provincial migration (Reference group)					
Inter-city in a province	0.183 *** (0.070)	0.213 *** (0.073)	−0.110 (0.087)	0.083 (0.105)	0.481 *** (0.071)
Inter-county in a city	0.183 *** (0.058)	0.195 *** (0.076)	−0.260 ** (0.130)	0.078 (0.071)	0.378 *** (0.067)
Duration in this city	0.034 *** (0.003)	0.034 *** (0.005)	0.027 *** (0.004)	0.040 *** (0.008)	0.037 *** (0.004)
Junior high school or lower (Reference group)					
High school	−0.160 *** (0.027)	−0.111 ** (0.045)	−0.135 *** (0.035)	−0.122 ** (0.048)	−0.175 *** (0.038)
College and higher	−0.286 *** (0.054)	−0.270 *** (0.054)	−0.356 *** (0.046)	−0.188 *** (0.071)	−0.269 *** (0.069)
Pension insurance	0.050 (0.056)	0.074 (0.069)	0.052 (0.057)	0.108 (0.077)	0.157 ** (0.061)
Economic strain	−0.248 *** (0.030)	−0.274 *** (0.041)	−0.236 *** (0.044)	−0.310 *** (0.049)	−0.248 *** (0.045)
Unemployed	−0.198 *** (0.069)	0.039 (0.135)	−0.141 * (0.073)	0.042 (0.157)	−0.277 ** (0.111)
Social inclusion	1.098 *** (0.052)	1.395 *** (0.10)	1.142 *** (0.071)	1.153 *** (0.077)	1.047 *** (0.090)
Obs.	96,930	17,065	43,707	30,700	39,588
Log-likelihood	−77,119.9	−13,947.6	−35,829.9	−23,969.8	−30,931.6
Pseudo-R^2^	0.029	0.034	0.027	0.033	0.038

Notes: All regressions also contain province dummy variables, not reported here for brevity. City-clustered robust standard errors are in parentheses for ordered logit regressions. * *p* < 0.10, ** *p* < 0.05, *** *p* < 0.01 (two-tailed z tests). The cell in bold below the standard error of the Income and Inequality coefficient reports the predicted change in the proportion of people in the bottom or top SWB category due to a one standard deviation change in the corresponding explanatory variable (see text for more details). The numbers in the brackets are 95% confidence intervals of effects sizes (please refer to the text for detailed information).

**Table 5:** Heterogeneous effects by income quartiles.

Dep Var: SWB	Lowest	Middle	Highest
	(1)	(2)	(3)
Income	0.317 *** (0.066) **0.0139** **[0.0099, 0.0178]**	0.176 *** (0.064) **0.0113** **[0.0057, 0.017]**	0.027 (0.036) **0.0029** [−**0.0025, 0.0083]**
Inequality	−1.577 *** (0.427) −**0.0137** **[**−**0.0193,**−**0.0081]**	−2.873 *** (0.625) −**0.0255** **[**−**0.0293,**−**0.0216]**	−4.556 *** (0.929) −**0.041** **[**−**0.0461,**−**0.0359]**
Other controls	Yes	Yes	Yes
Obs.	28,036	56,011	29,948
Log-likelihood	−22,211.3	−44,532.5	−24,260.7
Pseudo-R^2^	0.026	0.024	0.032

Notes: All regressions also contain province dummy variables, not reported here for brevity. City-clustered robust standard errors are in parentheses for ordered logit regressions. * *p* < 0.10, ** *p* < 0.05, *** *p* < 0.01 (two-tailed z tests). The cell in bold below the standard error of the Income and Inequality coefficient reports the predicted change in the proportion of people in the bottom or top SWB category due to a one standard deviation change in the corresponding explanatory variable (see text for more details). The numbers in the brackets are 95% confidence intervals of effects sizes (please refer to the text for detailed information). The results of the omitted controls are reported in Table A2.

**Table 6:** Income and inequality effects of migrants: additional results.

Dep Var: SWB	Controlling More Psychological Variables	Absolute Gini	OLS	CMDS 2012
Income	1.321 *** (0.249)	1.345 *** (0.251)	0.353 *** (0.065)	1.540 *** (0.226)
Income squared	−0.067 *** (0.015)	−0.068 *** (0.015)	−0.018 *** (0.004)	−0.013 * (0.006)
Inequality	−3.137 *** (0.265)	−0.365 *** (0.039)	−0.837 *** (0.242)	−3.126 *** (0.330)
Other controls	Yes	Yes	Yes	Yes
Obs.	113,995	113,995	113,995	156,962

Notes: All regressions also contain province dummy variables, not reported here for brevity. City-clustered robust standard errors are in parentheses. * *p* < 0.10, ** *p* < 0.05, *** *p* < 0.01 (two-tailed z tests). The results of the omitted controls are reported in Table A3.

**Table 7:** Results from heteroskedasticity based identification.

Dep Var: SWB	Lewbel (2012) 2-Stage Estimator	Lewbel (2012) GMM Estimator
Income	0.330 *** (0.143)	0.308 *** (0.104)
Income squared	−0.017 *** (−0.008)	−0.015 *** (0.006)
Inequality	−0.864 *** (0.246)	−0.784 *** (0.173)
Hansen’s *J*-Stat for overidentification		0.144
Kleibergen-Paap Wald *F*-Stat on excluded variables		51.049
Obs.	115,506	115,506

Notes: All regressions also contain the constant term, other variables-centered (12 controls, omitted) and province dummies, not reported here for brevity. The results of the omitted controls are reported in Table A4. Robust standard errors (in parentheses) for 2-stage and GMM estimator correct for clustering at the city level. *** *p* < 0.01 (two-tailed z tests).

**Table 8:** Social comparison concerns as a mechanism.

Dep Var: SWB	(1)	(2)	(3)	(4)
Income	1.374 *** (0.381)			1.161 *** (0.385)
Income squared	−0.080 *** (0.015)			−0.057 *** (0.016)
Relative income	0.166** (0.073)		1.037 *** (0.119)	0.746 *** (0.180)
Inequality		−3.087 *** (0.235)	−3.063 *** (0.246)	−3.102 *** (0.289)
Inequality ∗ Relative income		0.536 *** (0.083)	−2.333 *** (0.334)	−2.090 *** (0.353)
Other controls	Yes	Yes	Yes	Yes
Obs.	113,995	113,995	113,995	113,995
Log-likelihood	−91,119.1	−91,341.6	−91,214.5	−91,153.3
Pseudo-R^2^	0.027	0.028	0.029	0.029

Notes: All regressions also contain province dummy variables, not reported here for brevity. City-clustered robust standard errors are in parentheses for ordered logit regressions. *** *p* < 0.01 (two-tailed z tests). The results of the omitted controls are reported in Table A5.

**Table 9:** Expectations and perceived fairness serve as the channels of impact.

Dep Var: SWB	(1)	(2)	(3)
Income	1.317 *** (0.251)	1.310 *** (0.249)	1.317 *** (0.249)
Income squared	−0.066 *** (0.015)	−0.066 *** (0.015)	−0.066 *** (0.015)
Inequality	−3.209 *** (0.504)	−3.164 *** (0.371)	−3.148 *** (0.368)
I am concerned with changes of the city	1.348 *** (0.424)		
Inequality ∗ I am concerned with changes of the city	−1.986 *** (0.611)		
Migration distance		−0.761 *** (0.244)	
Inequality ∗ Migration distance		−2.674 *** (0.865)	
Perceived fairness			−0.593 ** (0.260)
Inequality ∗ Perceived fairness			−1.498 *** (0.343)
Other controls	Yes	Yes	Yes
Obs.	113,995	113,995	113,995
Log-likelihood	−90,974.4	−91,141.7	−90,801.3
Pseudo-R^2^	0.031	0.029	0.029

Notes: All regressions also contain province dummy variables, not reported here for brevity. City-clustered robust standard errors are in parentheses for ordered logit regressions. * *p* < 0.10, ** *p* < 0.05, *** *p* < 0.01 (two-tailed z tests). The results of the omitted controls are reported in Table A6.

## Appendix A

**Table A1:** Additional robust test results: putting “It is hard to say” answers together with “Unhappy”.

Dep Var: SWB.	Coefficient	Robust S.E.	*z*
Income	1.328	0.244	5.46 ***
Inequality	−3.644	0.247	−14.74 ***
Income squared	−0.065	0.014	−4.55 ***
Age	−0.025	0.006	−4.13 ***
Age squared/100	0.039	0.008	4.81 ***
Male	−0.059	0.016	−3.81 ***
Rural *hukou* status	0.193	0.025	7.60 ***
Han ethnicity	−0.310	0.046	−6.72 ***
Unmarried	0.000	0.030	0.01
Inter-provincial migration (Reference group)			
Inter-city in a province	0.216	0.032	6.73 ***
Inter-county in a city	0.197	0.041	4.80 ***
Duration in this city	0.030	0.002	12.95 ***
Junior high school or lower (Reference group)			
High school	−0.117	0.019	−6.09 ***
College and higher	−0.279	0.038	−7.31 ***
Pension insurance	0.065	0.036	1.82 *
Economic strain	−0.229	0.025	−9.27 ***
Unemployed	−0.171	0.061	−2.82 ***
Social inclusion	1.094	0.045	24.12 ***

Notes: Observations = 124,861. Ordered logit regression also contains province dummy variables, not reported here for brevity. Robust standard errors have been corrected for clustering at the city level. * *p* < 0.10, ** *p* < 0.05, *** *p* < 0.01 (two-tailed z tests).

## Appendix B

**Table A2:** The results of the omitted controls in Table 5.

Dep Var: SWB	Lowest	Middle	Highest
Age	−0.018 (0.011)	−0.035 *** (0.009)	−0.042 *** (0.011)
Age squared/100	0.033 ** (0.015)	0.056 *** (0.010)	0.065 *** (0.015)
Male	−0.082 *** (0.029)	−0.061 ** (0.024)	−0.020 (0.025)
Rural *hukou* status	0.119 * (0.063)	0.089 ** (0.045)	0.140 *** (0.035)
Han ethnicity	−0.442 *** (0.077)	−0.265 *** (0.068)	−0.240 *** (0.076)
Unmarried	−0.013 (0.053)	−0.016 (0.048)	−0.059 (0.060)
Inter-provincial migration (Reference group)			
Inter-city in a province	0.092 (0.083)	0.169 ** (0.072)	0.218 *** (0.075)
Inter-county in a city	0.053 (0.065)	0.160 ** (0.069)	0.294 *** (0.066)
Duration in this city	0.034 *** (0.005)	0.034 *** (0.003)	0.034 *** (0.005)
Junior high school or lower (Reference group)			
High school	−0.181 (0.039)	−0.123 *** (0.032)	−0.171 *** (0.043)
College and higher	−0.277 (0.063)	−0.254 *** (0.050)	−0.328 *** (0.058)
Pension insurance	0.055 (0.074)	0.049 (0.057)	0.107 (0.076)
Economic strain	−0.146 *** (0.042)	−0.239 *** (0.033)	−0.377 *** (0.038)
Unemployed	−0.146 (0.098)	−0.193 ** (0.084)	−0.060 (0.152)
Social inclusion	1.249 *** (0.077)	1.111 *** (0.064)	1.061 *** (0.070)

Notes: City-clustered robust standard errors are in parentheses for ordered logit regressions. * *p* < 0.10, ** *p* < 0.05, *** *p* < 0.01 (two-tailed z tests).

**Table A3:** The results of the omitted controls in Table 6.

Dep Var: SWB	Controlling More Psychological Variables	Absolute Gini	OLS	2012 CMDS
Age	−0.031 *** (0.007)	−0.032 *** (0.007)	−0.008 *** (0.002)	−0.109 *** (0.006)
Age squared/100	0.044 *** (0.010)	0.051 *** (0.010)	0.013 *** (0.003)	0.035 *** (0.007)
Male	−0.053 *** (0.017)	−0.053 *** (0.017)	−0.014 *** (0.005)	−0.064 *** (0.026)
Rural *hukou* status	0.128 *** (0.032)	0.114 *** (0.033)	0.031 *** (0.009)	0.175 *** (0.025)
Han ethnicity	−0.313 *** (0.064)	−0.321 *** (0.062)	−0.088 *** (0.016)	−0.279 *** (0.046)
Unmarried	−0.014 (0.036)	−0.012 (0.037)	−0.002 (0.009)	−0.020 (0.030)
Inter-provincial migration (Reference group)				
Inter-city in a province	0.152 ** (0.072)	0.164 ** (0.071)	0.046 ** (0.016)	0.213 *** (0.031)
Inter-county in a city	0.120 * (0.062)	0.162 *** (0.060)	0.047 *** (0.060)	0.190 *** (0.040)
Duration in this city	0.034 *** (0.003)	0.034 *** (0.003)	0.009 *** (0.001)	0.061 *** (0.010)
Junior high school or lower (Reference group)				
High school	−0.154 *** (0.025)	−0.152 *** (0.024)	−0.041 *** (0.007)	−0.107 *** (0.019)
College and higher	−0.295 *** (0.038)	−0.287 *** (0.037)	−0.080 *** (0.010)	−0.319 *** (0.058)
Pension insurance	0.074 (0.051)	0.072 (0.053)	0.012 (0.014)	0.064* (0.036)
Economic strain	−0.234 *** (0.027)	−0.253 *** (0.028)	−0.072 *** (0.008)	
Unemployed	−0.131 ** (0.066)	−0.131 ** (0.065)	−0.042 ** (0.017)	−0.182 *** (0.061)
Social inclusion	1.081 *** (0.050)	1.130 *** (0.047)	0.294 *** (0.012)	1.499 *** (0.046)
Psychological variable 1 (Attention to city change)	0.384 ** (0.166)			
Psychological variable 2 (Locals despise)	−0.463 *** (0.029)			

Notes: City-clustered robust standard errors are in parentheses for ordered logit regressions. * *p* < 0.10, ** *p* < 0.05, *** *p* < 0.01 (two-tailed z tests).

**Table A4:** The results of the omitted controls in Table 7.

Dep Var: SWB	Lewbel (2012) 2-Stage Estimator	Lewbel (2012) GMM Estimator
Age	−0.009 *** (0.002)	−0.007 *** (0.002)
Age squared/100	0.014 *** (0.003)	0.012 *** (0.002)
Male	−0.016 *** (0.005)	−0.012 *** (0.004)
Rural *hukou* status	0.024 *** (0.009)	0.089 ** (0.045)
Han ethnicity	−0.081 *** (0.016)	−0.092 *** (0.014)
Unmarried	−0.006 (0.011)	−0.005 (0.008)
Duration in this city	0.009 *** (0.001)	0.010 *** (0.001)
Inter-provincial migration	−0.025 *** (0.009)	−0.033 *** (0.007)
Junior high school or lower	0.036 *** (0.005)	−0.028 *** (0.004)
Pension insurance	0.012 (0.015)	0.010 (0.009)
Unemployed	−0.020 (0.014)	−0.022 * (0.012)
Social inclusion	0.298 *** (0.012)	0.293 *** (0.010)

Notes: City-clustered robust standard errors are in parentheses for ordered logit regressions. * *p* < 0.10, ** *p* < 0.05, *** *p* < 0.01 (two-tailed z tests).

**Table A5:** The results of the omitted controls in Table 8.

Dep Var: SWB	(1)	(2)	(3)	(4)
Age	−0.034 *** (0.007)	−0.029 *** (0.007)	−0.030 *** (0.007)	−0.031 *** (0.007)
Age squared/100	0.054 *** (0.010)	0.047 *** (0.010)	0.048 *** (0.010)	0.051 *** (0.010)
Male	−0.054 *** (0.017)	−0.049 *** (0.017)	−0.051 *** (0.017)	−0.052 *** (0.017)
Rural *hukou* status	0.115 *** (0.034)	0.117 *** (0.034)	0.115 *** (0.034)	0.112 *** (0.034)
Han ethnicity	−0.287 *** (0.070)	−0.308 *** (0.062)	−0.306 *** (0.062)	−0.325 *** (0.061)
Unmarried	−0.015 (0.036)	−0.044 (0.037)	−0.032 (0.038)	−0.008 (0.033)
Inter-provincial migration (Reference group)				
Inter-city in a province	0.163 ** (0.068)	0.158 ** (0.072)	0.156 ** (0.072)	0.169 *** (0.063)
Inter-county in a city	0.166 *** (0.063)	0.153 ** (0.061)	0.152 ** (0.061)	0.167 *** (0.057)
Duration in this city	0.034 *** (0.003)	0.034 *** (0.003)	0.034 *** (0.003)	0.034 *** (0.003)
Junior high school or lower (Reference group)				
High school	−0.163 *** (0.026)	−0.150 *** (0.025)	−0.152 *** (0.024)	−0.152 *** (0.024)
College and higher	−0.302 *** (0.038)	−0.283 *** (0.037)	−0.284 *** (0.036)	−0.285 *** (0.036)
Pension insurance	0.085 * (0.049)	0.081 (0.054)	0.079 (0.054)	0.063 (0.046)
Economic strain	−0.263 *** (0.029)	−0.258 *** (0.027)	−0.255 *** (0.028)	−0.252 *** (0.028)
Unemployed	−0.119 * (0.064)	−0.143 ** (0.064)	−0.132 ** (0.064)	−0.132 ** (0.062)
Social inclusion	1.132 *** (0.048)	1.128 *** (0.047)	1.128 *** (0.047)	1.131 *** (0.047)

Notes: City-clustered robust standard errors are in parentheses for ordered logit regressions. * *p* < 0.10, ** *p* < 0.05, *** *p* < 0.01 (two-tailed z tests).

**Table A6:** The results of the omitted controls in Table 9.

Dep Var: SWB	(1)	(2)	(3)
Age	−0.032 *** (0.007)	−0.032 *** (0.007)	−0.032 *** (0.007)
Age squared/100	0.052 *** (0.010)	0.051 *** (0.010)	0.050 *** (0.010)
Male	−0.054 *** (0.017)	−0.052 *** (0.017)	−0.053 *** (0.017)
Rural *hukou* status	0.114 *** (0.033)	0.111 *** (0.034)	0.124 *** (0.033)
Han ethnicity	−0.327 *** (0.061)	−0.313 *** (0.062)	−0.324 *** (0.063)
Unmarried	−0.006 (0.034)	−0.012 (0.035)	−0.016 (0.034)
Inter-provincial migration (Reference group)			
Inter-city in a province	0.168 ** (0.070)		0.148 ** (0.071)
Inter-county in a city	0.164 *** (0.060)		0.131 ** (0.062)
Duration in this city	0.034 *** (0.003)	0.034 *** (0.003)	0.035 *** (0.003)
Junior high school or lower (Reference group)			
High school	−0.155 *** (0.024)	−0.148 *** (0.024)	−0.158 *** (0.024)
College and higher	−0.291 *** (0.037)	−0.278 *** (0.037)	−0.302 *** (0.038)
Pension insurance	0.065 (0.053)	0.073 (0.054)	0.072 (0.052)
Economic strain	−0.250 *** (0.028)	−0.253 *** (0.028)	−0.231 *** (0.028)
Unemployed	−0.129 * (0.066)	−0.137 ** (0.067)	−0.125 * (0.065)
Social inclusion	0.987 *** (0.047)	1.131 *** (0.046)	1.081 *** (0.046)

Notes: City-clustered robust standard errors are in parentheses for ordered logit regressions. * *p* < 0.10, ** *p* < 0.05, *** *p* < 0.01 (two-tailed z tests).
